# Author Correction: Highly oxidized products from the atmospheric reaction of hydroxyl radicals with isoprene

**DOI:** 10.1038/s41467-025-59535-2

**Published:** 2025-05-01

**Authors:** Torsten Berndt, Erik H. Hoffmann, Andreas Tilgner, Hartmut Herrmann

**Affiliations:** Atmospheric Chemistry Department (ACD), Leibniz Institute for Tropospheric Research (TROPOS), 04318 Leipzig, Germany

**Keywords:** Atmospheric chemistry, Atmospheric chemistry

Correction to: *Nature Communications* 10.1038/s41467-025-57336-1, published online 28 February 2025

In the version of the article initially published, in the third paragraph of the “Detectable products for background NO level” section, the formula of compound **18** was “C_5_H_9_O_5_” but should have been “C_5_H_9_O_7_” and has now been corrected in the HTML and PDF versions of the article.

