# Supplementary material for: Highly oxidized products from the atmospheric reaction of hydroxyl radicals with isoprene
Source: Nat Commun. 2025 Feb 28;16:2068. doi: 10.1038/s41467-025-57336-1 (PMC11871199; doi:10.1038/s41467-025-57336-1)
Supplement: Supplementary file 1 — Supplementary Information [file 41467_2025_57336_MOESM1_ESM.pdf]

Supplementary Information for

Highly oxidized products from the atmospheric reaction of hydroxyl radicals with isoprene

Torsten Berndt[1]*, Erik H. Hoffmann[1], Andreas Tilgner[1] & Hartmut Herrmann[1]

[1] Atmospheric Chemistry Department (ACD), Leibniz Institute for Tropospheric Research (TROPOS), 04318 Leipzig, Germany.

Corresponding author: Torsten Berndt, e-mail: berndt@tropos.de

The PDF file includes:

Supplementary Figs. 1 to 19

Supplementary Tables S1 and S2

Supplementary References

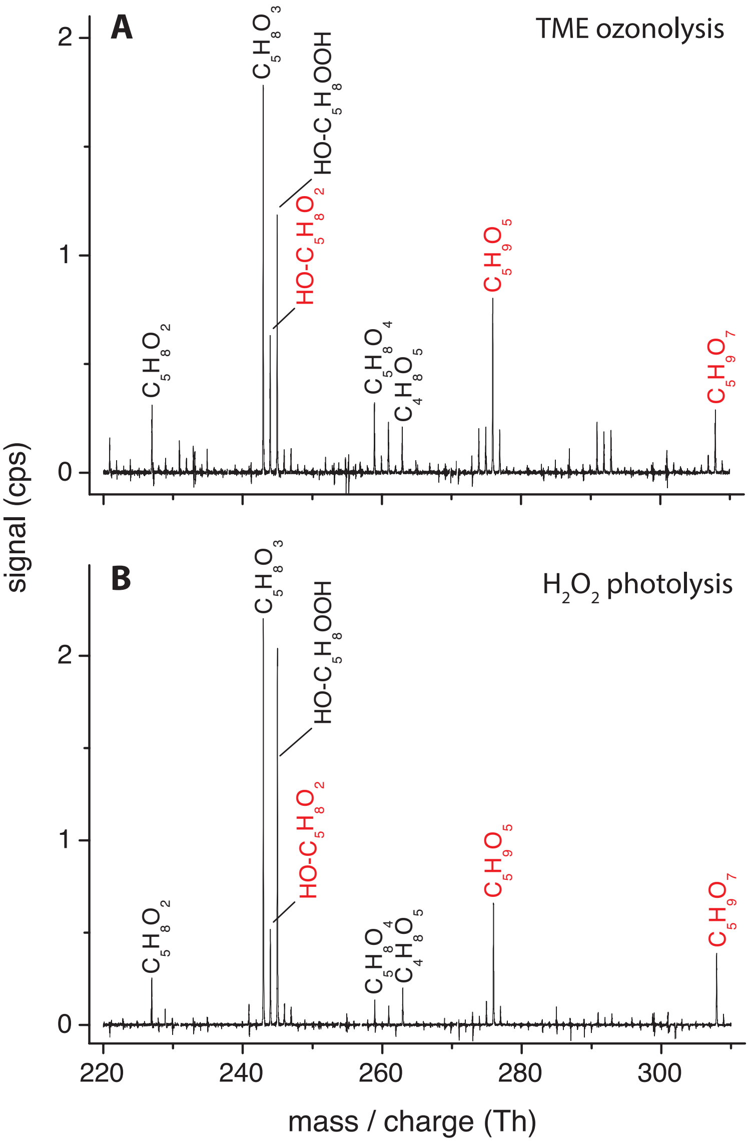

Supplementary Fig. 1: Product mass spectra from the OH + isoprene reaction. Spectra were recorded for an isoprene consumption of ~7×10^8 molecules cm^{-3} measured with a reaction time of 32 s in the LFT using ionization by iodide. Products appear as the adduct with iodide, their masses are shifted by 126.9 Th. Background NO concentration was $< 2 \times 10^8$ molecules cm^{-3}. HO_2 radical concentrations did not exceed 7×10^7 molecules cm^{-3} based on modeling results, see Methods. RO_2 radicals are indicated in red. The signal intensities do not reflect the product concentrations. OH radical formation via A) TME (tetramethylethylene) ozonolysis B) H_2O_2 photolysis.

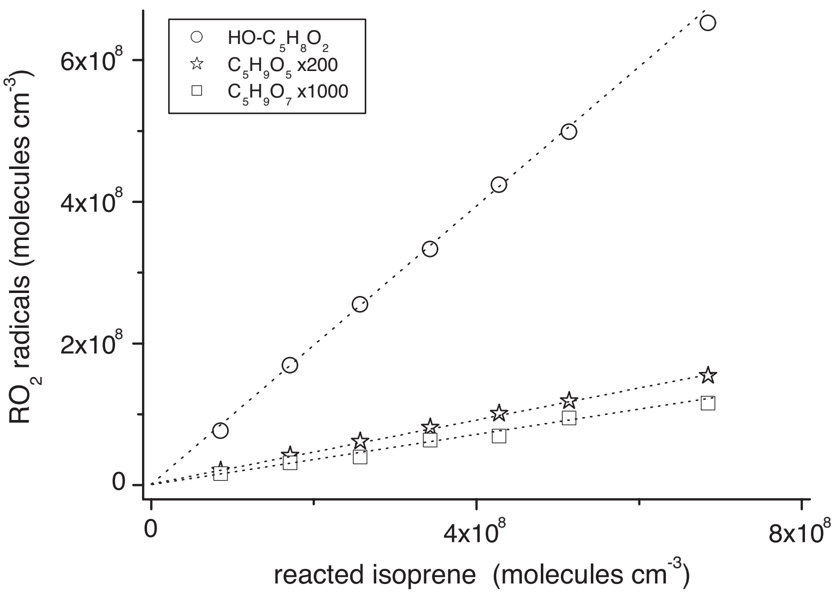

Supplementary Fig. 2: RO_2 radical concentrations as a function of converted isoprene measured for background NO conditions. Data were taken from the measurement series depicted in Fig. 4 of the main manuscript. Stated concentrations have an uncertainty of a factor of ~ 2. The error bars have been omitted for better clarity of the image.

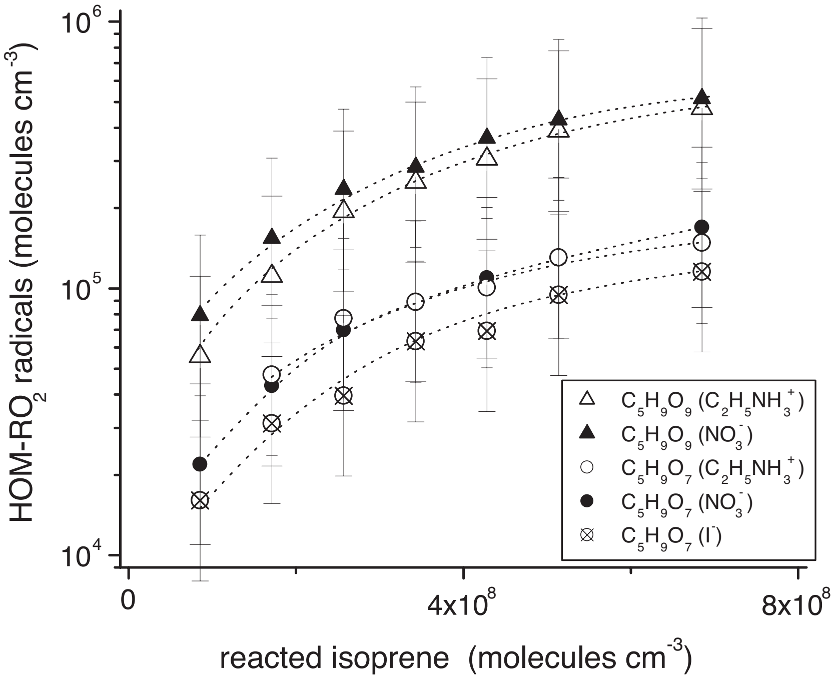

Supplementary Fig. 3: Formation of HOM-RO$_2$ radicals as a function of converted isoprene using different reagent ions. (HOM: highly oxygenated molecule) OH radicals were generated via TME (tetramethylethylene) ozonolysis. The NO concentration was $< 2 \times 10^8$ molecules cm^{-3} and the amount of reacted isoprene was calculated based on a comprehensive reaction mechanism, see Methods. The error bars represent the uncertainty of a factor of ~2. Reactant concentrations were [TME] = $(1.0 - 8.0) \times 10^{10}$, [isoprene] = $(1.25 - 10) \times 10^{11}$, and [$O_3$] = 3.0×10^{11} molecules cm^{-3}.

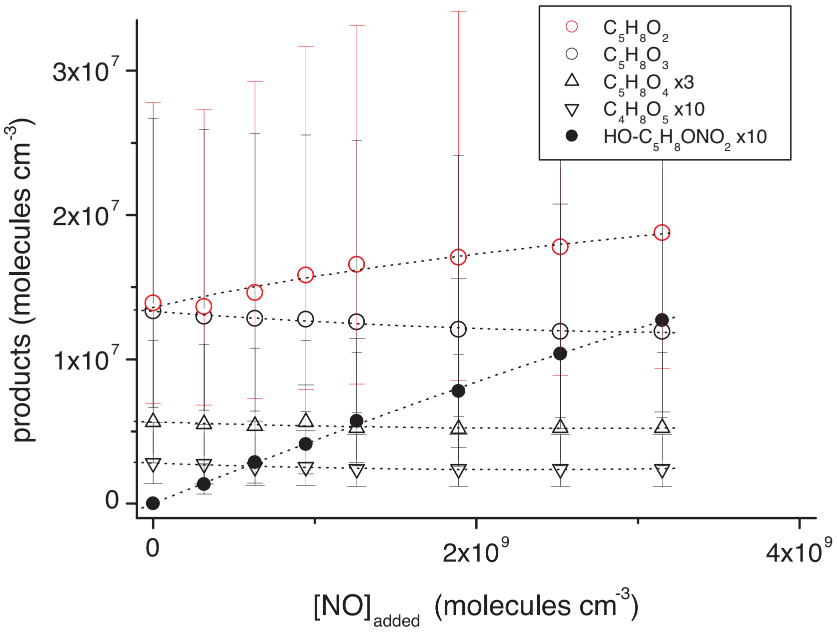

Supplementary Fig. 4: Changing product formation depending on added NO. Generation of OH radicals was carried out by TME (tetramethylethylene) ozonolysis using product analysis via iodide ionization. The error bars represent the uncertainty of a factor of ~ 2. Concentrations of $C_5H_8O_2$ and the organic nitrate, HO-$C_5H_8ONO_2$, are lower limit values. Reactant concentrations were: [TME] = 8.0×10^{10}, [isoprene] = 1.0×10^{12}, [O_3] = 3.0×10^{11}, and [NO] = $(3.2 - 32) \times 10^8$ molecules cm^{-3}.

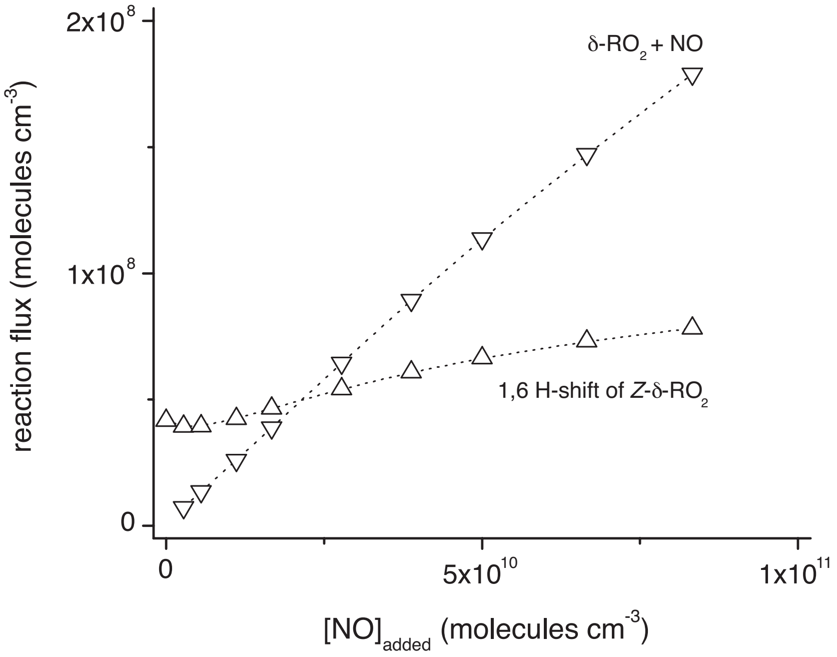

Supplementary Fig. 5: Reaction flux over the 1,6 H-shift channel forming allyl species 10 and the δ-RO_2 + NO channel forming 11 depending on added NO. Results from modeling for TME (tetramethylethylene) ozonolysis conditions with initial concentrations [TME] = 8.0×10^{10}, [isoprene] = 1.0×10^{12}, [O_3] = 3.0×10^{11} and added NO in the range of $(2.8 - 83) \times 10^9$ molecules cm^{-3}.

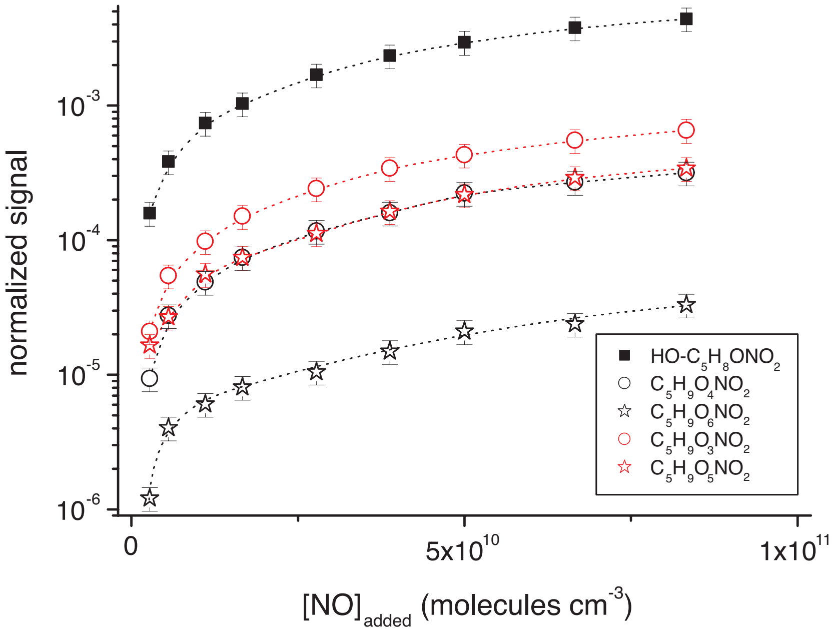

Supplementary Fig. 6: Organic nitrate formation as a function of added NO. Data were taken from the measurement series shown in Fig. 5 of the main manuscript. Organic nitrates formed via the allyl species **10** from 1,6 H-shift isomerization are given in open black symbols and those formed via **11** from δ-RO_2 + NO in open red symbols. Error bars represent the statistical uncertainty of ± 20 % from 10 min data accumulation.

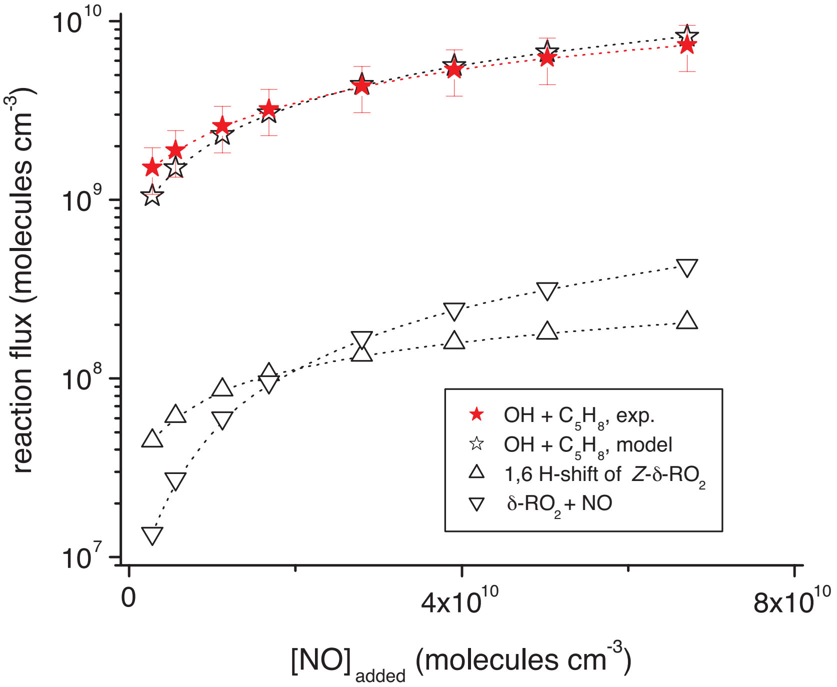

Supplementary Fig. 7: Comparison of converted isoprene from experiment and modeling and the reaction flux over the 1,6 H-shift and the δ-RO_2 + NO channel. Average OH radical concentrations in each experiment were measured by the SO_3 method adding a small concentration of SO_2 to the reaction gas, not influencing the desired OH + isoprene reaction, see Methods. Error bars represent the uncertainty of 29 % in the OH radical measurements. Results from modeling for IPN (isopropyl nitrite) photolysis conditions with initial concentrations [IPN] = 1.55×10^{11}, [isoprene] = 1.0×10^{12}, [SO_2] = 2.25×10^{12} and added NO in the range of $(2.8 - 67) \times 10^9$ molecules cm^{-3}.

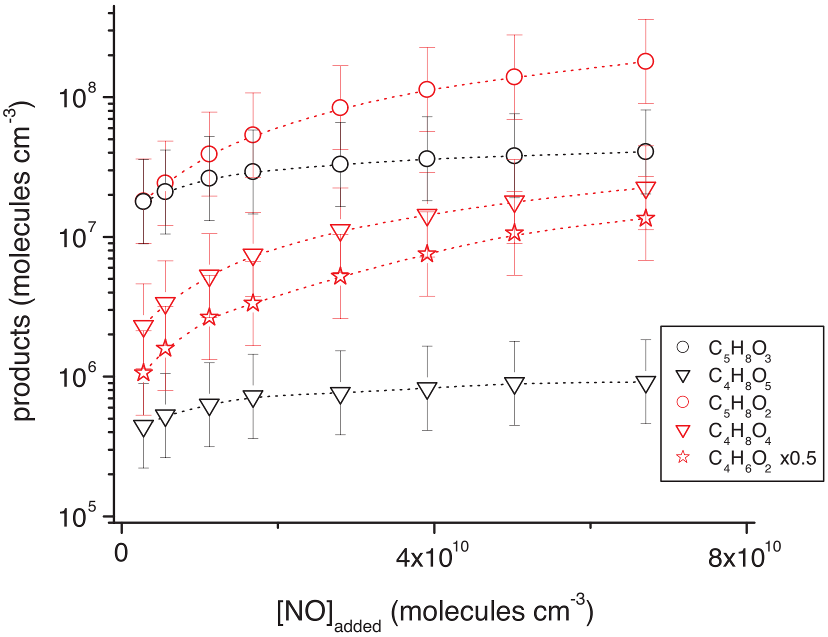

Supplementary Fig. 8: Closed-shell product formation as a function of added NO. Products mainly formed via the 1,6 H-shift channel are given in black and those from the δ-RO_2 + NO channel in red. IPN (isopropyl nitrite) photolysis served as the OH radical source and product analysis was carried out by iodide ionization. The error bars show the uncertainty of a factor of ~2. $C_5H_8O_2$ and $C_4H_6O_2$ concentrations are lower limit values. Reactant concentrations were [IPN] = 1.55×10^{11}, [isoprene] = 1.0×10^{12} and added NO was in the range of $(2.8 - 67) \times 10^9$ molecules cm^{-3}.

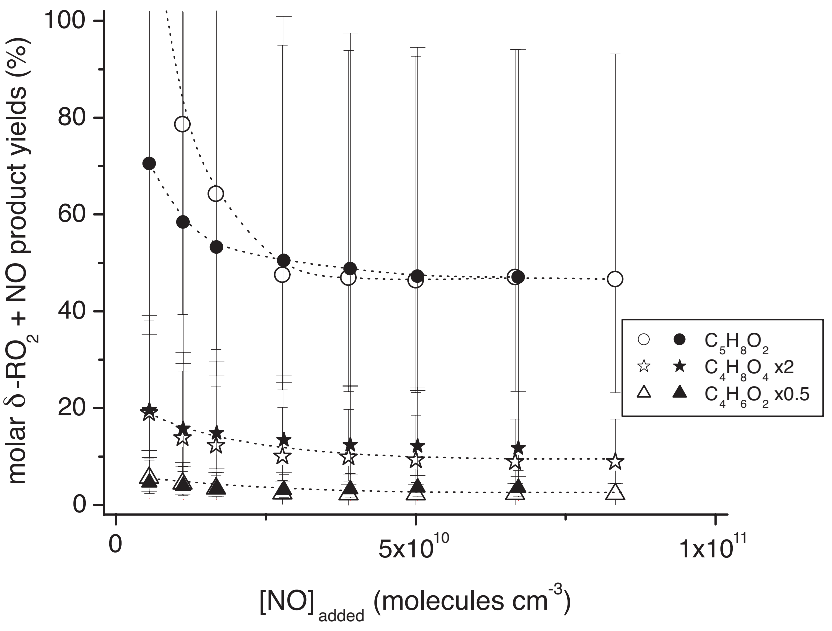

Supplementary Fig. 9: Molar product yields assigned to the δ-RO_2 + NO channel as a function of added NO. Data were taken from two measurement series using TME (tetramethylethylene) ozonolysis (open symbols) or IPN (isopropyl nitrite) photolysis (full symbols) for OH radical generation. Corresponding closed-shell product concentrations and reaction conditions are given in Fig. 5 and Supplementary Fig. 8, respectively. The reaction flux over the δ-RO_2 + NO channel was calculated using a comprehensive reaction mechanism, see Methods. Especially the measurement series with TME ozonolysis for OH generation indicates for low-NO conditions an additional pathway of $C_5H_8O_2$ formation leading to seeming molar yields > 100 %. However, all product yields showed an apparent increase with lowering of NO for [NO] < 3×10^{10} molecules cm^{-3}. Possible reasons are the relatively small product concentrations for these conditions affected with higher uncertainty and less accurate reaction flux calculated by the model. For [NO] > ~ 3×10^{10} molecules cm^{-3}, molar yields for $C_5H_8O_2$, $C_4H_8O_4$ and $C_4H_6O_2$ are ~ 47 %, ~ 5.5 % and ~ 4.5 %, respectively, with an uncertainty of a factor of ~ 2. The yields of $C_5H_8O_2$ and $C_4H_6O_2$ represent lower limit values.

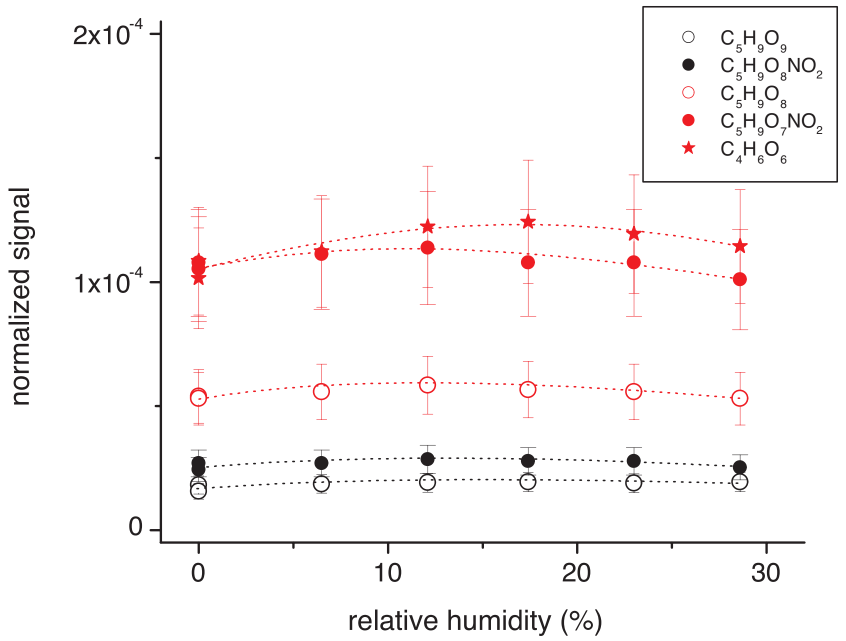

Supplementary Fig. 10: HOM product formation as a function of relative humidity r.h. (HOM: highly oxygenated molecule) OH radicals were formed via TME (tetramethylethylene) ozonolysis and the product analysis was carried out by nitrate ionization. Error bars show the uncertainty of the reagent ion-normalized product signals of ± 20 %. Small changes of product signals are probably due to the small changes in the reagent ion distribution with r.h., i.e., changing ratios of NO_3^- / $(HNO_3)NO_3^-$ / $(HNO_3)_2NO_3^-$, connected with a slightly different ionization efficiency of the different ions. Reactant concentrations were [TME] = 8.0×10^{10}, [isoprene] = 1.0×10^{12}, [O_3] = 3.0×10^{11} and [NO] = 6.7×10^{10} molecules cm^{-3}.

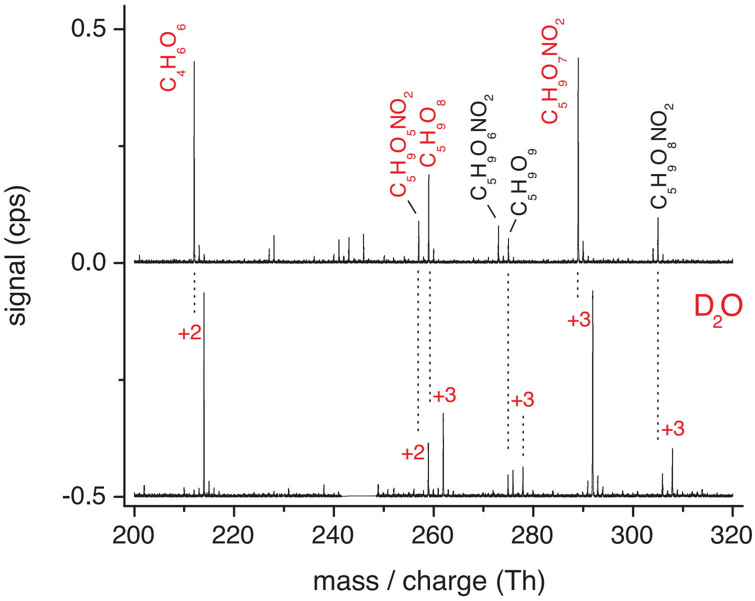

Supplementary Fig. 11: Mass spectra in the HOM range recorded in the absence and presence of heavy water, H/D-exchange experiments. (HOM: highly oxygenated molecule) 0.5 slm (STP) of D_2O saturated air was added to the reaction gas for H/D-exchange (lower part). OH radicals were generated by TME (tetramethylethylene) ozonolysis and the product analysis was carried out by nitrate ionization. The products appeared as the adduct with nitrate, i.e., their masses are shifted by 61.99 Th. Products assigned to the 1,6 H-shift channel are indicated in black and those from the δ-RO_2 + NO channel in red. The spectrum in the presence of D_2O is vertically moved by -0.5 units. In the case of $C_5H_9O_6NO_2$, a possible signal shift by 2 or 3 mass units can be concluded from the measurement, that indicates two different types of $C_5H_9O_7$ radicals bearing two or three OH and OOH groups in total. The latter could be formed from the $C_5H_9O_8$ + NO reaction after H-shift from an OOH group in the resulting alkoxy radical. Reactant concentrations were [TME] = 8.0×10^{10}, [isoprene] = 1.0×10^{12}, [O_3] = 3.0×10^{11} and [NO] = 6.7×10^{10} molecules cm^{-3}.

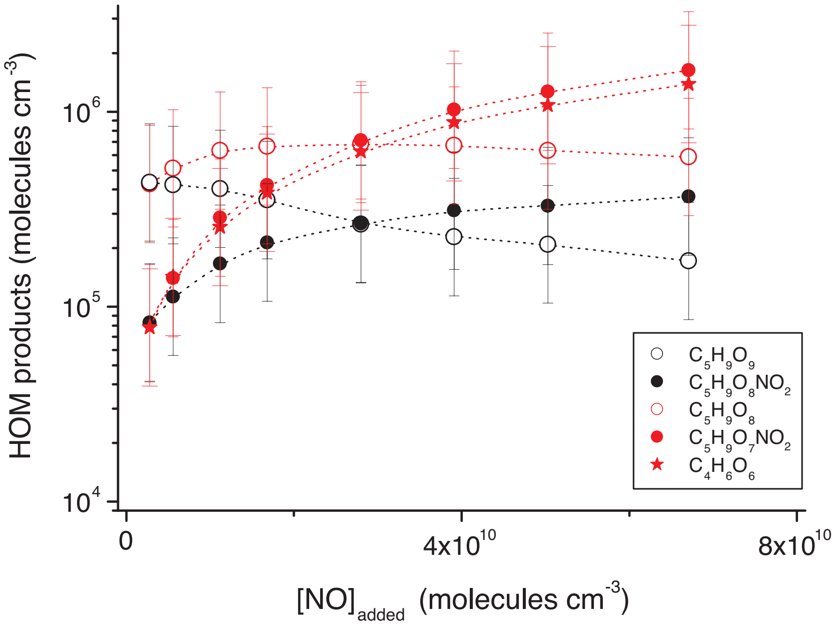

Supplementary Fig. 12: Most important HOM products as a function of added NO. (HOM: highly oxygenated molecule) Products from the 1,6 H-shift channel are given in black and those from the δ-RO_2 + NO channel in red. OH radicals were produced from IPN (isopropyl nitrite) photolysis and the product analysis was carried out by nitrate ionization. Error bars show the uncertainty of product concentrations of a factor of ~2. Reactant concentrations were [IPN] = 1.55×10^{11}, [isoprene] = 1.0×10^{12} and added NO was in the range of $(2.8 - 67) \times 10^9$ molecules cm^{-3}.

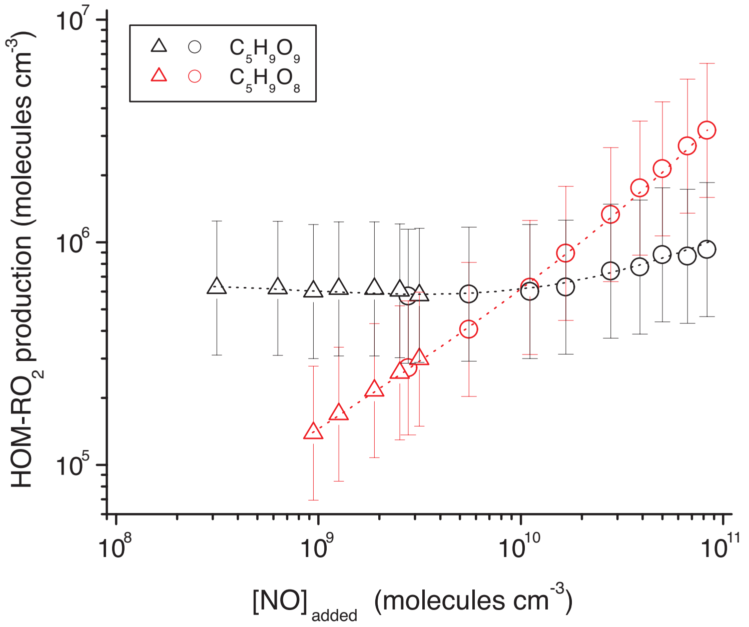

Supplementary Fig. 13: Amount of produced HOM-RO_2 radicals $C_5H_9O_8$ and $C_5H_9O_9$ depending on added NO. (HOM: highly oxygenated molecule) Measured HOM-RO_2 concentrations were taken from experiments using TME (tetramethylethylene) ozonolysis for OH radical generation as given in Fig. 7 (circles) and Supplementary Fig. 4 (triangles), but here using nitrate ionization for product detection. The amount of formed "final" HOM-RO_2 radical concentrations were calculated via equation (13) (Methods). Error bars represent the uncertainty of a factor of ~2 in the measured RO_2 concentrations.

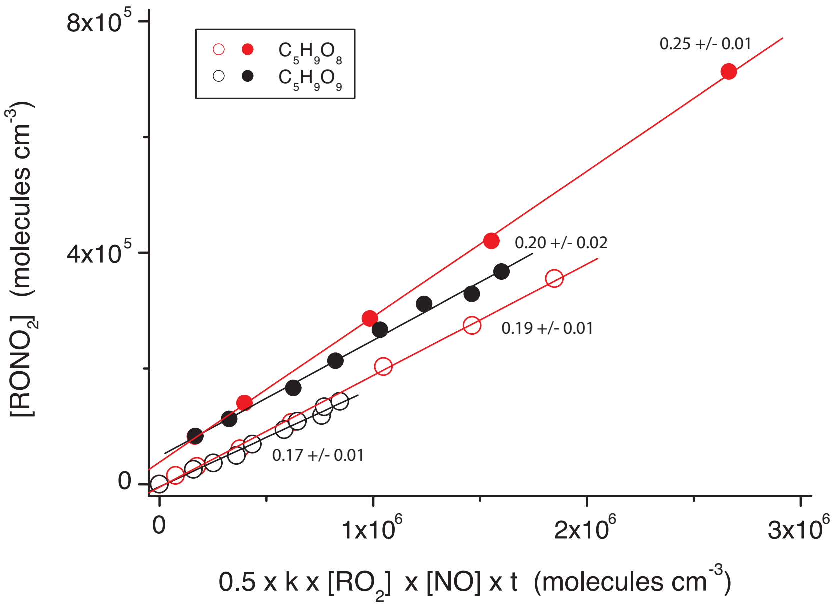

Supplementary Fig. 14: Analysis of organic nitrate formation from $C_5H_9O_8$ and $C_5H_9O_9$ according to equation (15). Open symbols show the results from the experiments with TME (tetramethylethylene) ozonolysis for OH generation (Fig. 7) and closed symbols from IPN (isopropyl nitrite) photolysis (Supplementary Fig. 12). Reported values represent the slopes including the statistical uncertainty. Resulting molar $RONO_2$ formation yield from both measurement series each is 22 ± 4 % for $C_5H_9O_8$ and 19 ± 3 % for $C_5H_9O_9$ considering the statistical uncertainty only.

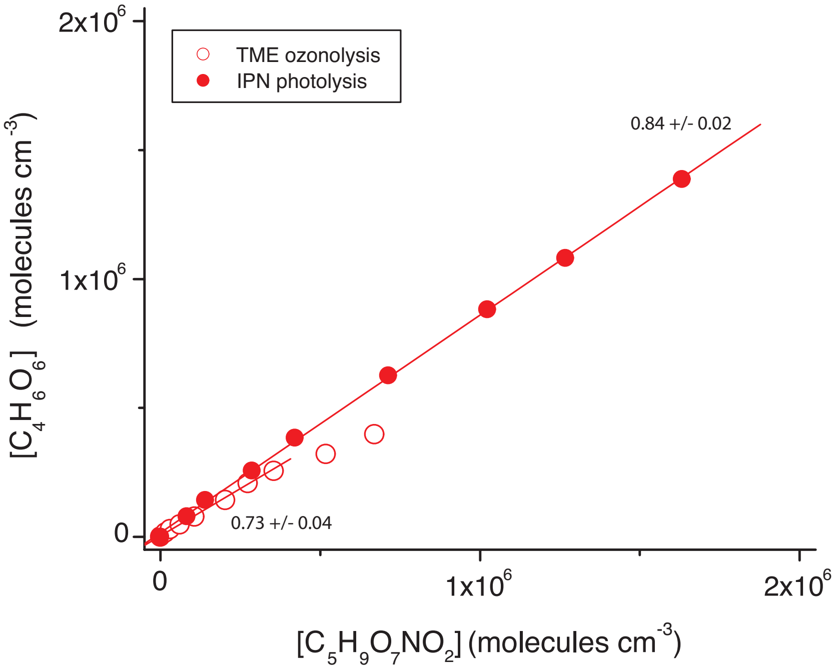

Supplementary Fig. 15: Ratio of the concentrations of $C_4H_6O_6$ and $C_5H_9O_7NO_2$ from two measurement series. (TME: tetramethylethylene, IPN: isopropyl nitrite) Experimental data were taken from the measurement series given in Fig. 7 and Supplementary Fig. 12. The two highest values from the experiments with TME ozonolysis for OH radical generation were omitted in the analysis. Resulting product ratio $[C_4H_6O_6] / [C_5H_9O_7NO_2]$ from both measurement series is 0.78 ± 0.09.

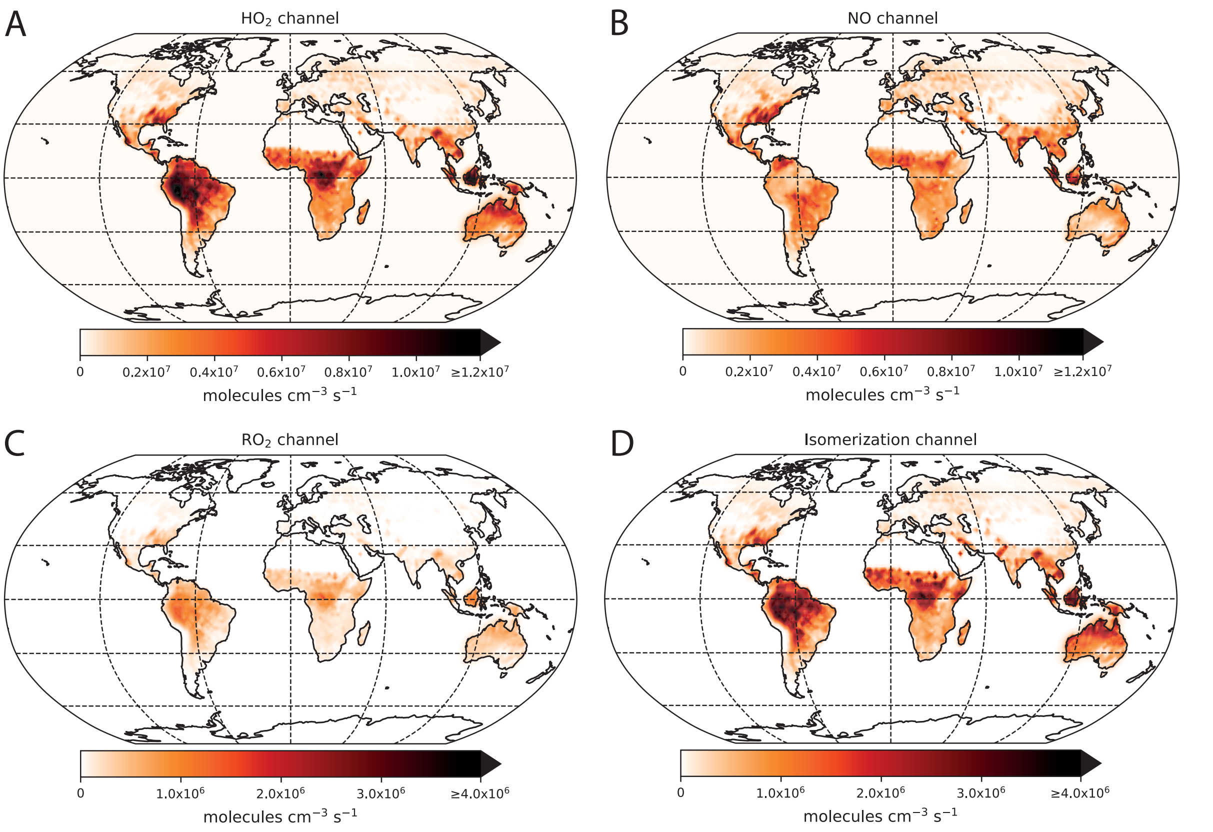

Supplementary Fig. 16: Spatial distribution of the annual mean HO-$C_5H_8O_2$ reaction rates. Shown are the vertical sum for each grid cell regarding the mean reaction rate of HO-$C_5H_8O_2$ radicals with **A** HO_2 radicals, **B** NO, **C** RO_2 radicals (CH_3O_2 and $CH_3C(O)O_2$) or **D** via 1,6 H-shift isomerization. The data were plotted with Python using the packages cartopy (projection: Robinson)[1] and matplotlib (https://matplotlib.org/).[2,3]

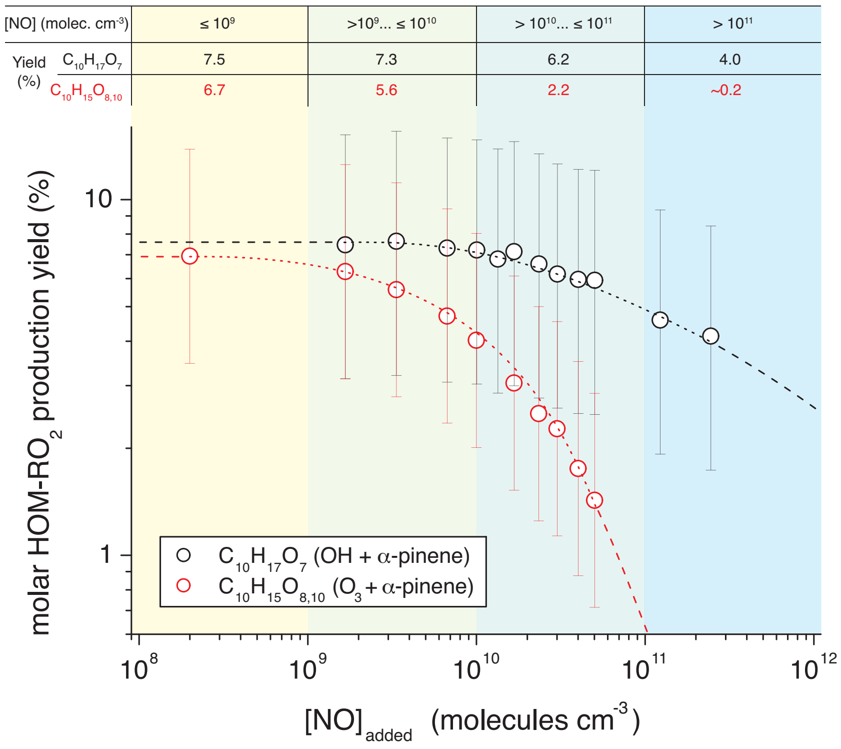

Supplementary Fig. 17: Molar production yields of the HOM-RO$_2$ radicals $C_{10}H_{15}O_{8,10}$ and $C_{10}H_{17}O_7$ depending on NO concentration. (HOM: highly oxygenated molecule) HOM-RO$_2$ measurements for α-pinene ozonolysis were taken from the experiments shown in Figure 5 in reference[4] considering both $C_{10}H_{15}O_8$ and $C_{10}H_{15}O_{10}$ in total, here marked as $C_{10}H_{15}O_{8,10}$. The production of $C_{10}H_{15}O_{8,10}$ was calculated according to equation (SV) in reference[4], identically equal to equation (13) of the present study, using k(RO$_2$+NO) = 8.8×10^{-12} cm^3 molecule^{-1} s^{-1}, T = 295 K. For OH + α-pinene, the needed HOM-RO$_2$ measurements were taken from the experiments depicted in Figure 2a in reference[5]. The corresponding $C_{10}H_{17}O_7$ production was calculated according to equation (SXII) in reference[5], again identically equal to equation (13) of the present study, using also k(RO$_2$+NO) as mentioned before. In the normalization with respect to converted α-pinene, non-converted initially formed RO$_2$ radicals, i.e., $C_{10}H_{15}O_4$ from the ozonolysis[4] and $C_{10}H_{17}O_3$ from the OH radical reaction[5], have been considered. Error bars represent the uncertainty of a factor of ~2 in the measured HOM-RO$_2$ concentrations. The measurement point at [NO] = 2×10^8 molecules cm^{-3} in the case of α-pinene ozonolysis represents the production yield for background NO conditions. The dashed lines show assumed extrapolations. Stated average yields for given NO ranges were used in the global modeling.

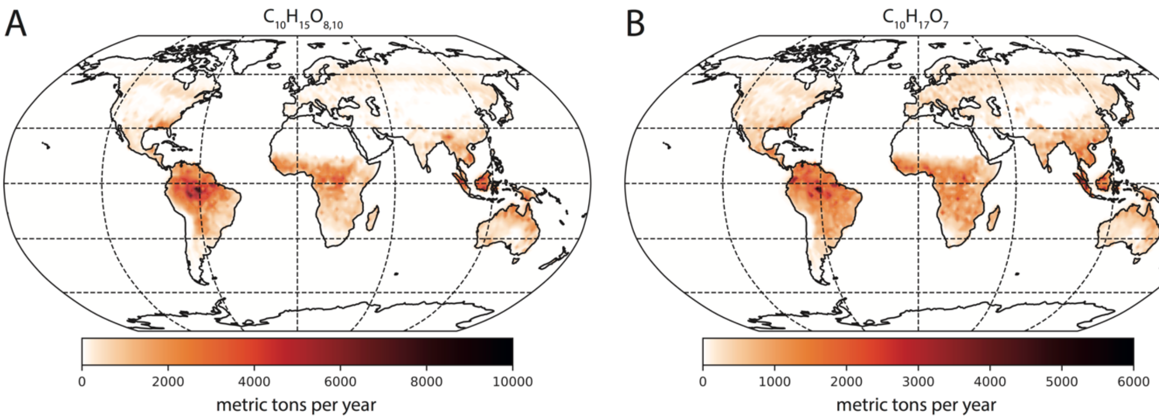

Supplementary Fig. 18: Spatial distribution of HOM-RO_2 radical production from O_3/OH + α-pinene. Annual total production in each grid cell for **A** $C_{10}H_{15}O_{8,10}$ from the ozonolysis of α-pinene and **B** $C_{10}H_{17}O_7$ from OH + α-pinene. The data were plotted with Python using the packages cartopy (projection: Robinson)[1] and matplotlib (https://matplotlib.org/).[2,3]

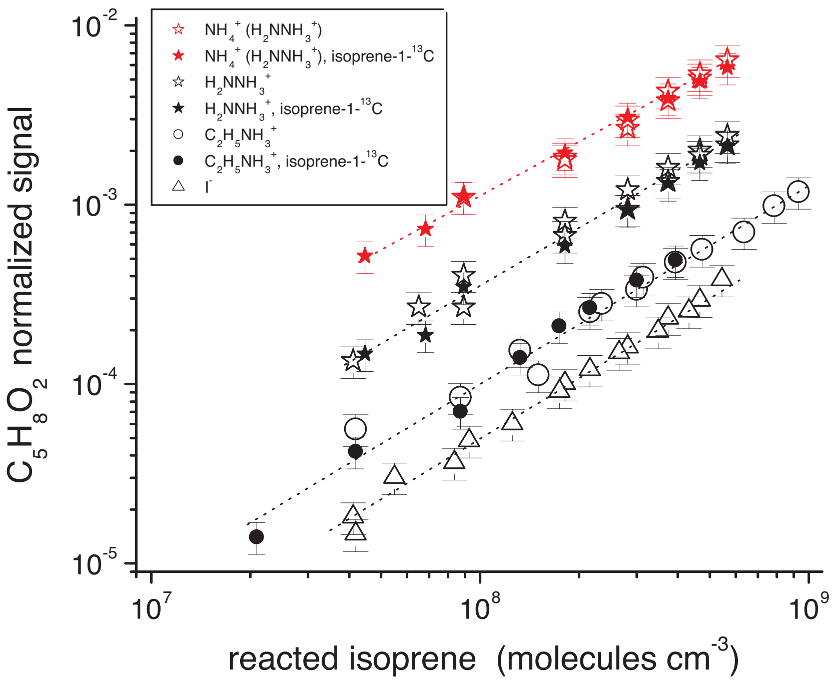

Supplementary Fig. 19: Normalized $C_5H_8O_2$ signal as a function of converted isoprene for different ionization schemes. The experiments were conducted in the free-jet flow system[6] with a reaction time of 7.9 s. OH radicals were formed via TME (tetramethylethylene) ozonolysis. Unlabeled and ^{13}C-labeld isoprene was used in the experiments, which showed identical results. The error bars represent the maximum statistical uncertainty of ±20 % from 10 min data accumulation. Data observed by means of hydrazinium ionization were taken from re-analysis of former experiments.[6] In the case of ammonium ionization, direct determination of normalized signal intensity was impossible because the ammonium signal at nominal 18 Th could not be measured with the needed reliability. Here, the $C_5H_8O_3$ signal measured by ammonium and hydrazinium ionization was taken as internal standard assuming $C_5H_8O_3$ detection with close to maximum sensitivity in both cases, see Figure 4c in reference[6]. Reactant concentrations were in the range: [TME] = (1.0 - 2.0) × 10^{11}, [isoprene] = (1.25 - 2.5) × 10^{12}, [isoprene-1-^{13}C] = (1.9 - 2.5) × 10^{12} and [O_3] = (1.5 - 97) × 10^{10} molecules cm^{-3}.

Supplementary Table 1. Reaction scheme of OH + isoprene describing the processes in the flow experiments. Rate coefficients at 295 K were taken from literature or have been estimated. Radicals arising from the OH attack in 1-position are marked with "I" and those from 4-position with "II".

Reaction	Rate coefficient (cm^3 molecule^{-1} s^{-1} or s^{-1})
O_3 + TME → 0.40×sCI + 0.52×OH + 0.08×HO_2 + 0.52×$CH_3C(O)CH_2O_2$	1.0×10^{-15} [7]
sCI → OH + $CH_3C(O)CH_2O_2$	900 [8]
IPN (+O_2) → HO_2 + NO + acetone	0.0007
OH + TME → products	1.1×10^{-10} [9]
NO + HO_2 → OH + NO_2	8.9×10^{-12} [9]
OH + HO_2 → H_2O + O_2	1.1×10^{-10} [9]
HO_2 + HO_2 → H_2O_2 + O_2	1.65×10^{-12} [9]
OH + NO → HNO_2	1.0×10^{-11} [9]
OH + NO_2 → HNO_3	1.2×10^{-11} [9]
O_3 + NO → NO_2 + O_2	1.8×10^{-14} [9]
O_3 + NO_2 → NO_3 + O_2	3.5×10^{-17} [9]
O_3 + HO_2 → OH + 2 × O_2	2.0×10^{-15} [9]
O_3 + isoprene → 0.26×OH + 0.26 × HO_2 + products	1.3×10^{-17} [10]
OH + isoprene → 0.315×trans OH-adduct I + 0.315×cis OH-adduct I + 0.111×trans OH-adduct II + 0.259×cis OH-adduct II	1.0×10^{-10} [10,11]
trans OH-adduct I + O_2 → *E*-δ-HO-RO_2 I	3.2×10^{-13} [11]
E-δ-HO-RO_2 I → trans OH-adduct I + O_2	13 [11]
trans OH-adduct I + O_2 → β-HO-RO_2 I	7.8×10^{-13} [11]
β-HO-RO_2 I → trans OH-adduct I + O_2	1.26 [11]
cis OH-adduct I + O_2 → β-HO-RO_2 I	7.8×10^{-13} [11]
β-HO-RO_2 I → cis OH-adduct I + O_2	0.226 [11]
cis OH-adduct I + O_2 → *Z*-δ-HO-RO_2 I	1.2×10^{-13} [11]
Z-δ-HO-RO_2 I → cis OH-adduct I + O_2	17.9 [11]
Z-δ-HO-RO_2 I → 1,6 H-shift	0.29 [11]
β-HO-RO_2 I → 1,5 H-shift	0.0
trans OH-adduct II + O_2 → *E*-δ-HO-RO_2 II	4.9×10^{-13} [11]
E-δ-HO-RO_2 II → trans OH-adduct II + O_2	3.0 [11]

trans OH-adduct II + O_2 → β-HO-RO_2 II	7.1×10^{-13}, [11]
β-HO-RO_2 II → trans OH-adduct II + O_2	0.23 [11]
cis OH-adduct II + O_2 → β-HO-RO_2 II	7.1×10^{-13}, [11]
β-HO-RO_2 II → cis OH-adduct II + O_2	0.11 [11]
cis OH-adduct II + O_2 → *Z*-δ-HO-RO_2 II	2.1×10^{-13}, [11]
Z-δ-HO-RO_2 II → cis OH-adduct II + O_2	8.2 [11]
Z-δ-HO-RO_2 II → 1,6 H-shift	3.4 [11]
β-HO-RO_2 II → 1,5 H-shift	0.0
E-δ-HO-RO_2 I + HO_2 → ROOH + O_2	1.74×10^{-11}, [10]
β-HO-RO_2 I + HO_2 → ROOH + O_2	1.74×10^{-11}, [10]
Z-δ-HO-RO_2 I + HO_2 → ROOH + O_2	1.74×10^{-11}, [10]
E-δ-HO-RO_2 II + HO_2 → ROOH + O_2	1.74×10^{-11}, [10]
β-HO-RO_2 II + HO_2 → ROOH + O_2	1.74×10^{-11}, [10]
Z-δ-HO-RO_2 II + HO_2 → ROOH + O_2	1.74×10^{-11}, [10]
$CH_3C(O)CH_2O_2$ + HO_2 → ROOH + O_2	9.0×10^{-12}, [12]
E-δ-HO-RO_2 I + NO → 0.87×1,4-OHcarb + 0.87×HO_2 + products<br>→ δ-RO_2 + NO product	8.8×10^{-12}, [10]
β-HO-RO_2 I + NO → 0.87×1,2-OHcarb + 0.87×HO_2 + products	8.8×10^{-12}, [10]
Z-δ-HO-RO_2 I + NO → 0.87×1,4-OHcarb + 0.87×HO_2 + products<br>→ δ-RO_2 + NO product	8.8×10^{-12}, [10]
E-δ-HO-RO_2 II + NO → 0.87×4,1-OHcarb + 0.87×HO_2 + products<br>→ δ-RO_2 + NO product	8.8×10^{-12}, [10]
β-HO-RO_2 II + NO → 0.87×4,3-OHcarb + 0.87×HO_2 + products	8.8×10^{-12}, [10]
Z-δ-HO-RO_2 II + NO → 0.87×4,1-OHcarb + 0.87×HO_2 + products<br>→ δ-RO_2 + NO product	8.8×10^{-12}, [10]
$CH_3C(O)CH_2O_2$ + NO → 0.87×HO_2 + products	8.8×10^{-12}, [10]
OH → wall	0.053 [a]
HO_2 → wall	0.045 [a]
E-δ-HO-RO_2 I → wall	0.016 [a]
β-HO-RO_2 I → wall	0.016 [a]
Z-δ-HO-RO_2 I → wall	0.016 [a]
E-δ-HO-RO_2 II → wall	0.016 [a]

β-HO-RO_2 II → wall	0.016 [a]
Z-δ-HO-RO_2 II → wall	0.016 [a]
$CH_3C(O)CH_2O_2$ → wall	0.016 [a]

[a] diffusion-limited rate coefficient estimated for the LFT.

Supplementary Table 2. Estimated calibration factors according to a convergence method. The stated calibration factors for product ionization with ethylaminium $C_2H_5NH_3^+$ and iodide I^- are based on a factor f for maximum sensitivity of 2×10^9 molecules cm^{-3} affected with an uncertainty of a factor of ~2.

Product	$f(C_2H_5NH_3^+$, product) (molecules cm^{-3})	$f(I^-$, product) (molecules cm^{-3})
HO-$C_5H_8O_2$ [a]	~3×10^{11}	~1.6×10^{12}
$C_5H_9O_5$	$(4\ ^{+4}_{-2}) \times 10^9$	$(2\ ^{+2}_{-1}) \times 10^9$
$C_5H_9O_7$ [b]	$(2\ ^{+2}_{-1}) \times 10^9$	$(2\ ^{+2}_{-1}) \times 10^9$
$C_5H_9O_9$ [b]	$(2\ ^{+2}_{-1}) \times 10^9$	–
$C_5H_8O_3$	$(5\ ^{+5.0}_{-2.5}) \times 10^9$	$(1\ ^{+1.0}_{-0.5}) \times 10^{10}$
$C_5H_8O_4$	$(5\ ^{+5.0}_{-2.5}) \times 10^9$	$(1\ ^{+1.0}_{-0.5}) \times 10^{10}$
$C_4H_8O_5$	$(4\ ^{+4}_{-2}) \times 10^9$	$(3\ ^{+3.0}_{-1.5}) \times 10^9$
$C_5H_8O_2$ [c]	$(1.8\ ^{+1.8}_{-0.9}) \times 10^{10}$	$(5\ ^{+5.0}_{-2.5}) \times 10^{10}$
$C_4H_8O_4$ [d]	$(4\ ^{+4}_{-2}) \times 10^9$	$(3\ ^{+3.0}_{-1.5}) \times 10^9$
$C_4H_6O_2$ [c,e]	$(1.8\ ^{+1.8}_{-0.9}) \times 10^{10}$	$(5\ ^{+5.0}_{-2.5}) \times 10^{10}$

[a] rough estimation [b] maximum sensitivity assumed [c] lower limit value
[d] assumed to be the same as for $C_5H_8O_4$ [e] assumed to be the same as for $C_5H_8O_2$

Supplementary references

1 Met Office. Cartopy: a cartographic python library with a Matplotlib interface (https://scitools.org.uk/cartopy). (2010-2015).

2 Hunter, J. D. Matplotlib: A 2D Graphics Environment. *Comput. Sci. Eng.* **9**, 90-95, doi:10.1109/mcse.2007.55 (2007).

3 Hunter, J., Dale, D., Firing, E., Droettboom, M. & the Matplotlib development team. Matplotlib v3.8.0. doi:10.5281/zenodo.8347255 (2012-2025).

4 Berndt, T. Peroxy Radical and Product Formation in the Gas-Phase Ozonolysis of α-Pinene under Near-Atmospheric Conditions: Occurrence of an Additional Series of Peroxy Radicals $O,O-C_{10}H_{15}O(O_2)_yO_2$ with y = 1-3. *J. Phys. Chem. A* **126**, 6526-6537, doi:10.1021/acs.jpca.2c05094 (2022).

5 Berndt, T. Peroxy Radical Processes and Product Formation in the OH Radical-Initiated Oxidation of α-Pinene for Near-Atmospheric Conditions. *J. Phys. Chem. A* **125**, 9151-9160, doi:10.1021/acs.jpca.1c05576 (2021).

6 Berndt, T., Hyttinen, N., Herrmann, H. & Hansel, A. First oxidation products from the reaction of hydroxyl radicals with isoprene for pristine environmental conditions. *Commun. Chem.* **2**, 21, doi:10.1038/s42004-019-0120-9 (2019).

7 Witter, M., Berndt, T., Böge, O., Stratmann, F. & Heintzenberg, J. Gas-phase ozonolysis: Rate coefficients for a series of terpenes and rate coefficients and OH yields for 2-methyl-2-butene and 2,3-dimethyl-2-butene. *Int. J. Chem. Kinet.* **34**, 394-403, doi:10.1002/kin.10063 (2002).

8 Peltola, J., Seal, P., Vuorio, N., Heinonen, P. & Eskola, A. Solving the discrepancy between the direct and relative-rate determinations of unimolecular reaction kinetics of dimethyl-substituted Criegee intermediate $(CH_3)_2COO$ using a new photolytic precursor. *Phys. Chem. Chem. Phys.* **24**, 5211-5219, doi:10.1039/d1cp02270a (2022).

9 Atkinson, R. *et al.* Evaluated Kinetic and Photochemical Data for Atmospheric Chemistry: Supplement IV. IUPAC Subcommittee on Gas Kinetic Data Evaluation for Atmospheric Chemistry. *J. Phys. Chem. Ref. Data* **21**, 1125-1568, doi:10.1063/1.555918 (1992).

10 Wennberg, P. O. *et al.* Gas-Phase Reactions of Isoprene and Its Major Oxidation Products. *Chem. Rev.* **118**, 3337-3390, doi:10.1021/acs.chemrev.7b00439 (2018).

11 Teng, A. P., Crounse, J. D. & Wennberg, P. O. Isoprene peroxy radical dynamics. *J. Am. Chem. Soc.* **139**, 5367-5377, doi:10.1021/jacs.6b12838 (2017).

12 Atkinson, R. *et al.* Evaluated Kinetic, Photochemical and Heterogeneous Data for Atmospheric Chemistry: Supplement V. IUPAC Subcommittee on Gas Kinetic Data Evaluation for Atmospheric Chemistry. *J. Phys. Chem. Ref. Data* **26**, 521-1011, doi:10.1063/1.556011 (1997).
